# Supplementary material for: Cell environment shapes TDP-43 function with implications in neuronal and muscle disease
Source: Commun Biol. 2022 Apr 5;5:314. doi: 10.1038/s42003-022-03253-8 (PMC8983780; doi:10.1038/s42003-022-03253-8)
Supplement: Supplementary file 2 — Supplementary Information [file 42003_2022_3253_MOESM2_ESM.pdf]

Suppl. figure 1

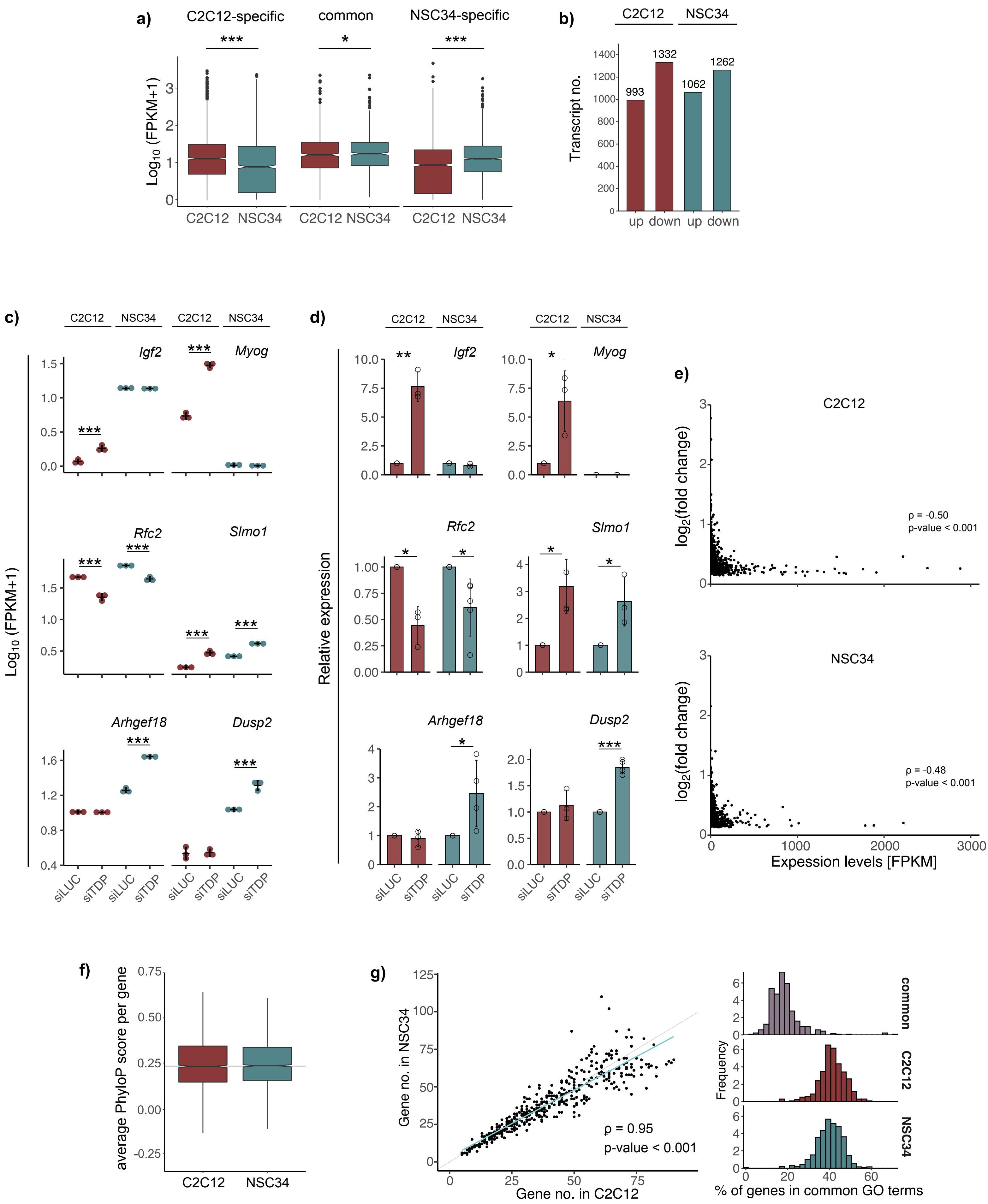

Supplementary figure 1. DEGs detected in C2C12 and NSC34. a) The plot shows log10-transformed FPKM values of muscular, neuronal and common TDP-43 targets in siLUC-transfected C2C12 and NSC34 cells. C2C12-specific DEGs exhibit higher expression in C2C12 cells (p-value $< 2.2\cdot10^{-16}$), while NSC34-specific DEGs have higher expression in NSC34 (p-value $< 2.2\cdot10^{-16}$). Expression levels of common targets is more similar between cell lines (p-value = 0.02). Significance was tested using Wilcoxon signed-rank test. **b)** The diagram shows the number of upregulated and downregulated genes detected in C2C12 and NSC34 cells following TDP-43 silencing. **c)** Expression changes of representative DEGs (C2C12-specific vs. common vs. NSC34-specific, Fig. 2d) as assessed by RNA-seq and plotted as log_{10}-transformed FPKM. **d)** Relative expression changes of DEGs from Supplementary Fig. 1c were validated using qPCR. p-values were generated using Student's t test (paired, one-tailed, n ≥ 3 per group). **e)** Scatter plots show there is no correlation between the absolute change in gene expression following TDP-43 depletion (plotted as log_2-transformed fold change) and the baseline expression of a given transcript (FPKM in siLUC-transfected cells) for DEGs identified in C2C12 (2325) and NSC34 (2324) (Spearman's $\rho = -0.50$, p-value $< 2.2\cdot10^{-16}$ and Spearman's $\rho = -0.48$, p-value $< 2.2\cdot10^{-16}$, respectively). **f)** Average per gene PhyloP conservation scores plotted as box plots show TDP-43-regulated DEGs detected in C2C12 (2325) and NSC34 (2324) are equally well conserved across species (p-value = 0.48). p-value was generated using Wilcoxon rank sum test, the grey line represents the median of average PhyloP scores of all exons in the mouse genome. **g)** The number of DEGs found in commonly enriched GO terms (Supplementary Fig. 2e, 459) is similar between two cell lines (left). Grey line represents y = x and the blue line the fitted regression (Spearman's $\rho = 0.95$, p-value $< 2.2\cdot10^{-16}$). Frequency plot shows that commonly regulated terms are highly enriched for cell-type-specific TDP-43-regulated DEGs (right).

Suppl. figure 2

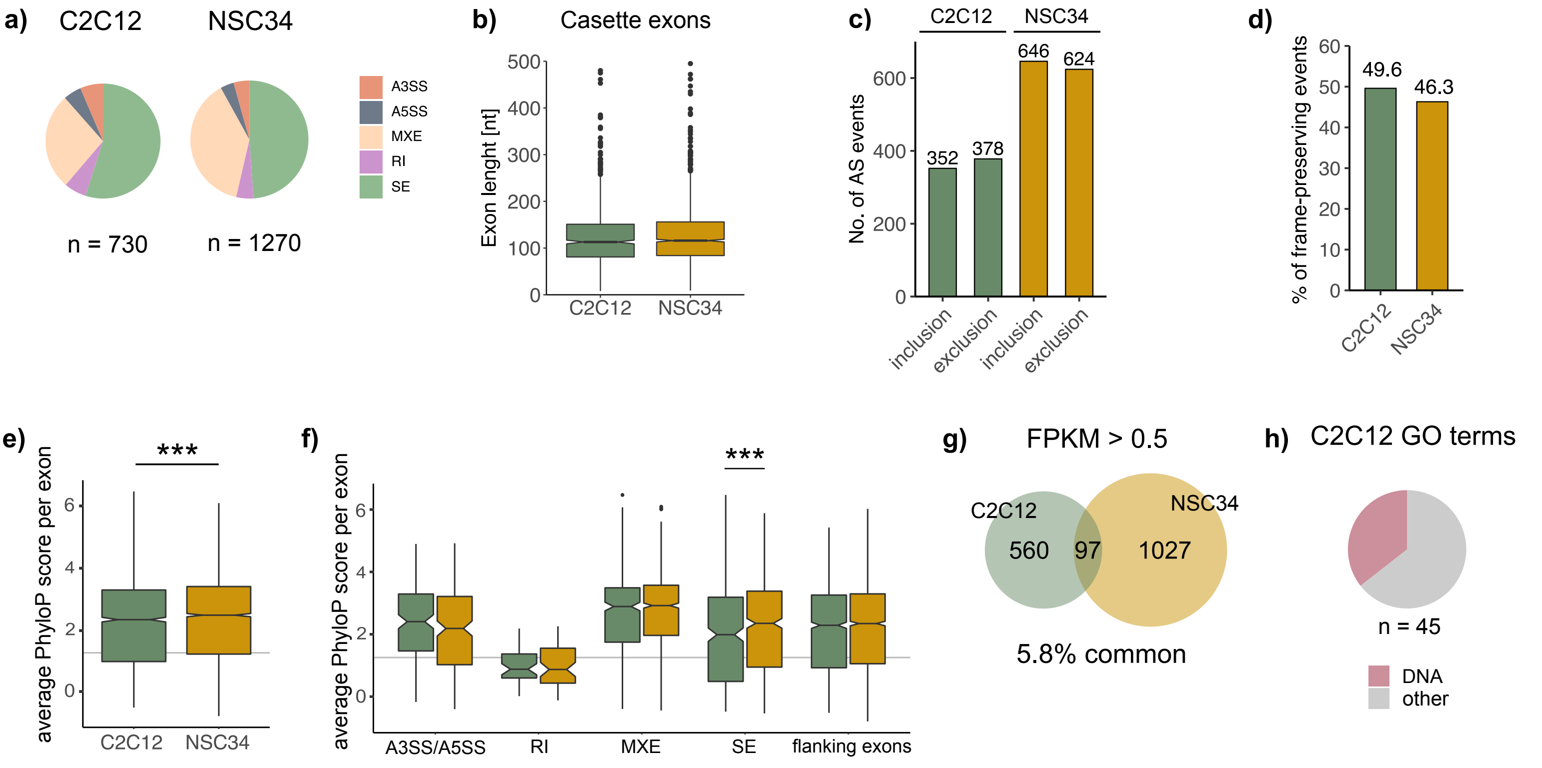

Supplementary figure 2. General features of TDP-43-controlled AS events detected in C2C12 and NSC34. a) TDP-43-regulated AS events detected in C2C12 and NSC34 cells do not differ in terms of event type distribution (the number below shows the total number of AS events detected in each cell line); **b)** the average length of TDP-43-regulated cassette exons (SE and MXE); **c)** the ratio between inclusion/exclusion events; **d)** the percentage of frame-conserving events. **e)** Average per exon PhyloP conservation scores plotted as box plots show TDP-43-regulated alternative sequences detected in NSC34 cells (4281) are better conserved across species than those detected in C2C12 cells (2372) (p-value = $1.1 \cdot 10^{-4}$). p-value was generated using Wilcoxon rank sum test with continuity correction, the grey line represents the median of average PhyloP scores of all exons in the mouse genome. **f)** Average per exon PhyloP conservation scores of TDP-43-regulated alternative sequences stratified by event type (SE, MXE, RI, A3'SS, A5'SS). The difference (p-value = $6.5 \cdot 10^{-6}$) among all groups was tested with Kruskal-Wallis rank sum test, followed by pairwise comparisons using Wilcoxon rank sum test with Benjamini-Hochberg correction for multiple testing. Significant difference is highlighted only for within event comparison between two tissues, SE (p-value = $9.7 \cdot 10^{-3}$). The grey line represents the median of average PhyloP scores of all exons in the mouse genome. **g)** Venn diagram shows the total number of AS events (detected by rMATS as in (Fig. 3a)) occurring in transcripts, which are expressed in both cell lines (FPKM in both cell lines > 0.5). **h)** GO enrichment analysis (refers to Fig. 3g) was performed on alternatively spliced genes detected in C2C12 using less stringent threshold for genes which entered GO analysis (p-value < 0.01 instead of FDR < 0.01). Resulting GO terms (45) imply on dysregulation of DNA-related biological processes.

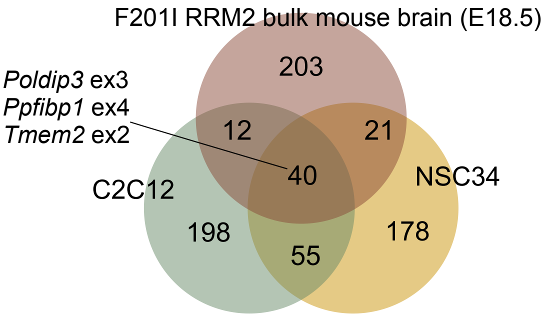

Supplementary figure 3. TDP-43-regulated splicing changes in mouse cell lines and bulk brain tissue. Venn diagram shows the number of AS events (junctions) detected by MAJIQ (ΔPSI > 0.2, FDR < 0.1) detected in C2C12 and NSC34 cell lines (as in Fig. 3b) together with those identified in brain tissue of mice carrying a mutation in the RNA-recognition motif RRM2 (F210I) of endogenous *Tardbp*. That RNA-seq dataset was generated by *Fratta et al* (2018).

Suppl. figure 4

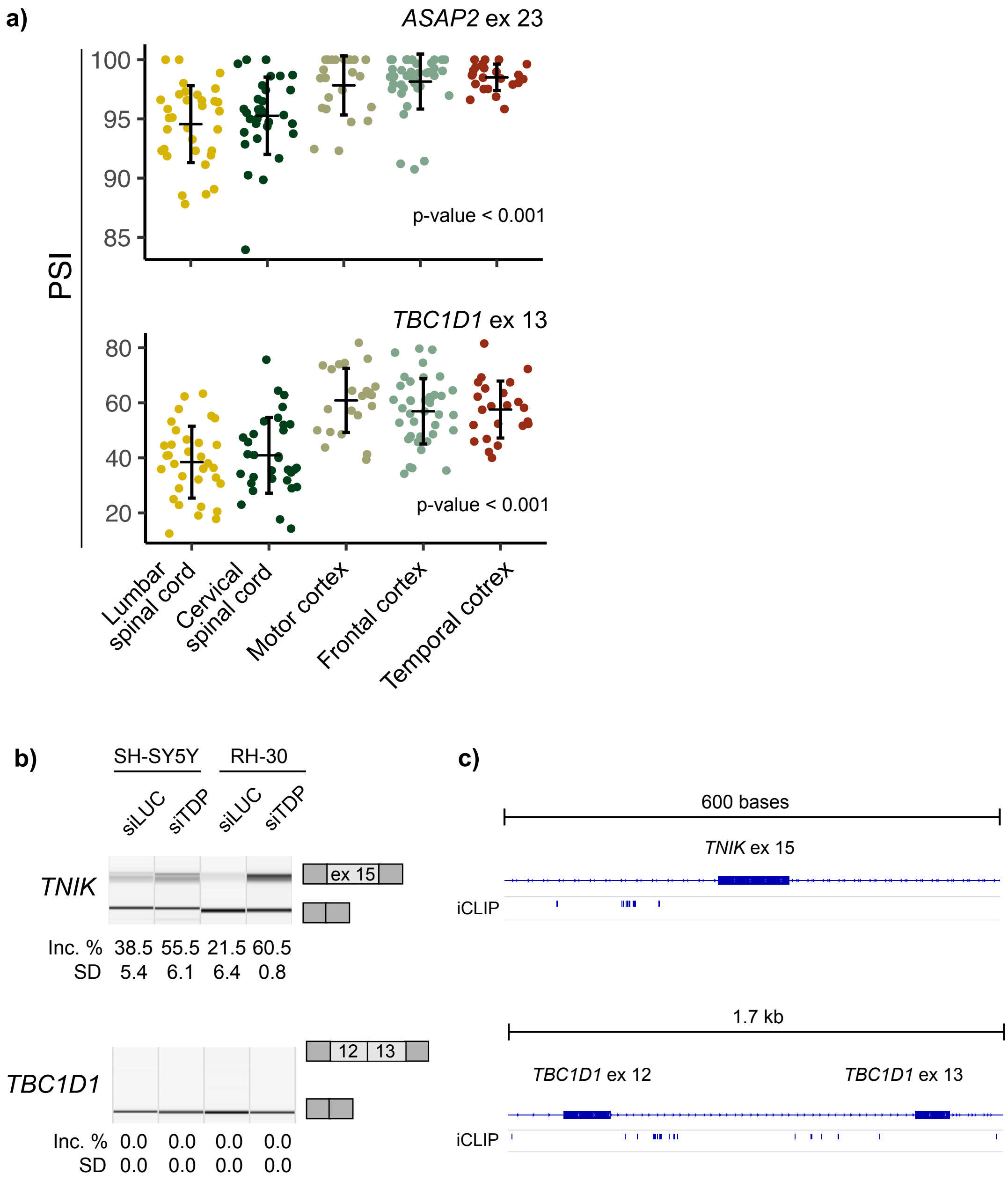

Supplementary figure 4. Tissue-characteristic inclusion of alternative exons regulated by TDP-43. a) Dot plots demonstrate variable inclusion levels of alternative exons across different brain regions of healthy controls (in the absence of TDP-43 pathology), as exemplified by two alternative exons – exon 23 of *ASAP2* and exon 13 of *TBC1D1*. p-values (p-value = $1.6 \cdot 10^{-9}$ for *ASAP2* and p-value = $2.4 \cdot 10^{-11}$ for *TBC1D1*, respectively) were generated using Kruskal-Wallis chi-squared test. **b)** Exon 15 of human *TNIK* (orthologous to exon 14 in mouse (Fig. 6b)) is regulated by TDP-43 in both, SH-SY5Y and RH-30 cell line, likely in a direct fashion by TDP-43 binding in the upstream intron as shown in Supplementary Fig. 4c. The long isoform of *TBC1D1* gene (exons 12 and 13 included) is not expressed in undifferentiated SH-SY5Y and RH-30 cells (orthologous event in mouse (Fig. 6b)), as inclusion of exons 12 and 13 increases with differentiation (*Bland et al*, 2010), however, TDP-43 binding sites were identified in the vicinity of exons represented in Supplementary Fig. 4c. Semi quantitative RT-PCRs conducted in TDP-43-silenced cells and corresponding controls are shown along with the quantification of splicing changes (% of alternative exon inclusion) (see the exact transcript numbers in Supplementary Table 2, n = 3 replicates per group). **c)** Schematic representation of TDP-43 binding sites identified by iCLIP analysis in SH-SY5Y cells (*Tollervey et al*, 2011) in the vicinity of exons represented in Supplementary Fig. 4b.

Suppl. figure 5

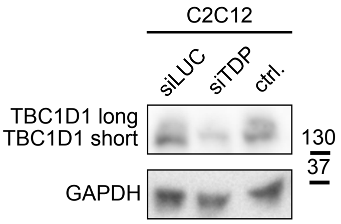

Supplementary figure 5. TDP-43 dependent expression of TBC1D1 isoforms. Both Tbc1d1 mRNA isoforms detected in C2C12 cells (Fig. 6b) are translated into protein. Western blot shows expression of both TBC1D1 protein isoforms (128 and 138 kDa, respectively) in siLUC-transfected or untreated (ctrl.) C2C12 cells, while only the smaller protein isoform is expressed in TDP-43-silenced cells. The amount of TBC1D1 was normalized against GAPDH or tubulin.

Suppl. figure 6

a) *Fig. 1a*

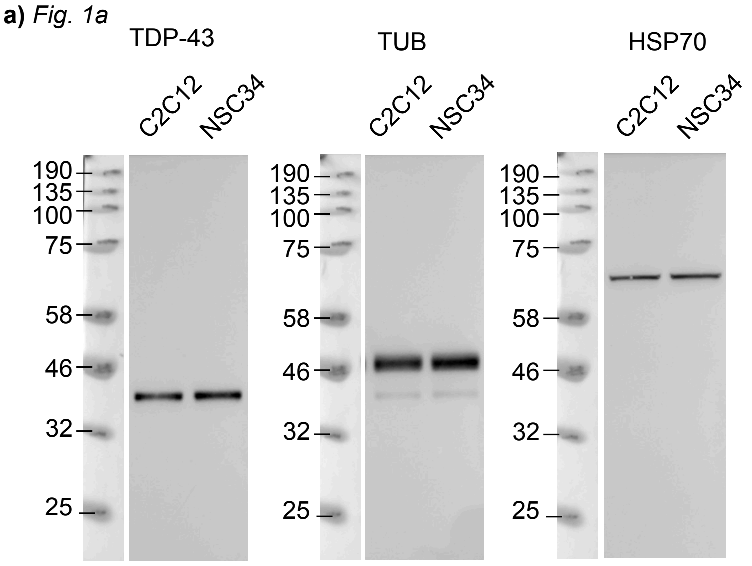

b) *Fig. 1c C2C12*

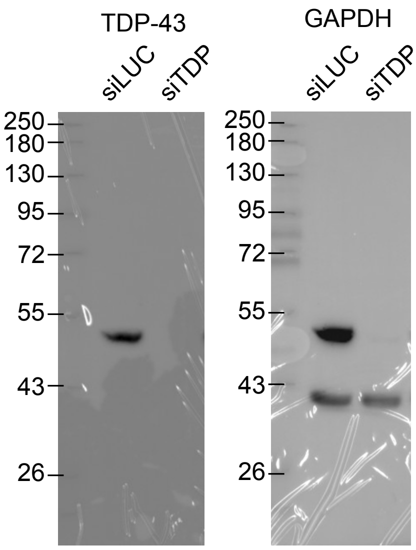

c) *Fig. 1c NSC34*

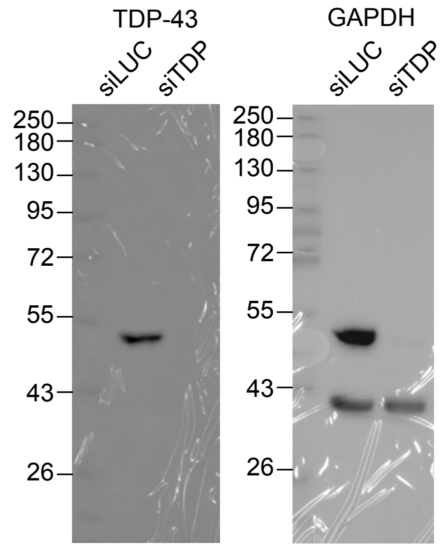

d) *Fig. 8a SH-SY5Y*

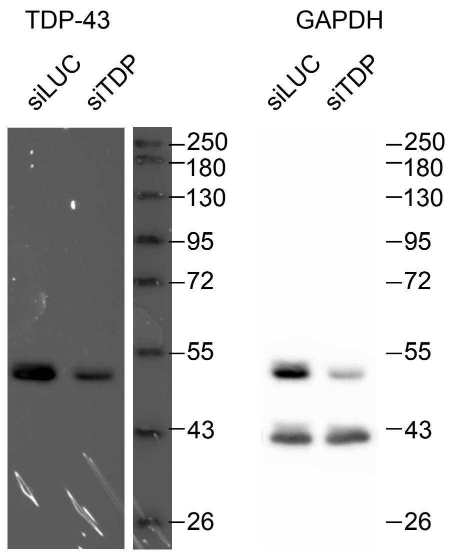

e) *Fig. 8a RH-30*

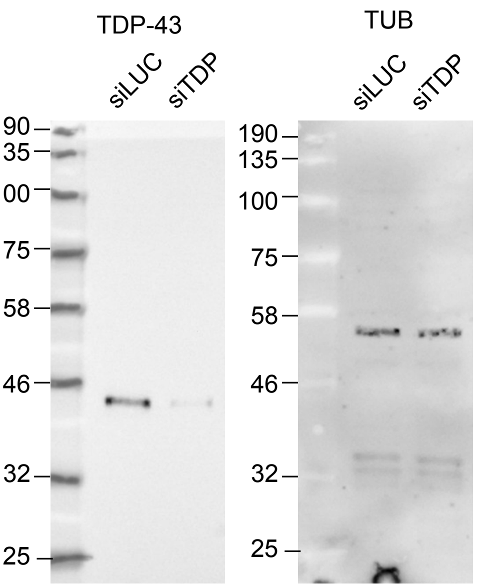

f) *Suppl. Fig. 5*

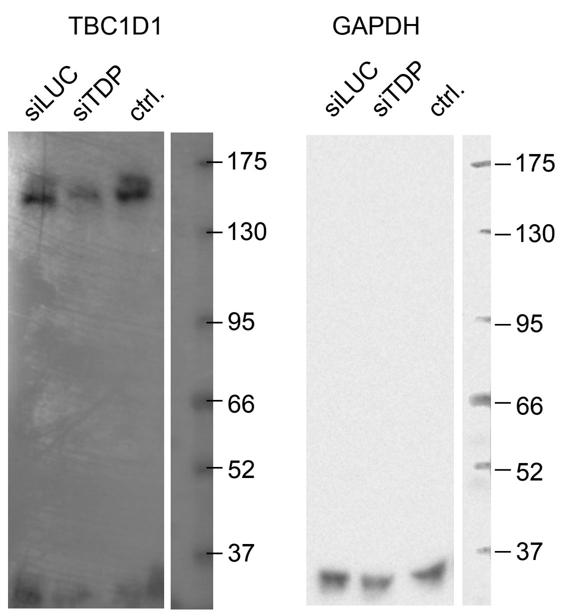

Supplementary figure 6. Uncropped and unedited blot images. **a)** Western blot (Fig. 1a) shows similar expression of endogenous TDP-43 in C2C12 and NSC34 cells. The amount of TDP-43 was normalized to the sum of peak intensities of three loading controls (tubulin, HSP70 and P84) (n = 3 replicates per group). **b)** and **c)** Western blots (Fig. 1c) show the reduction of TDP-43 in C2C12 and NSC34 cells upon siTDP transfection. siLUC-transfected cells were used as a control. TDP-43 expression was normalized against GAPDH (n = 3 replicates per group). **d)** and **e)** Western blots (Fig. 8a) show efficient reduction of TDP-43 in SH-SY5Y and RH-30 cells upon siTDP transfection. The amount of TDP-43 was normalized against GAPDH or tubulin (n = 3 replicates per group). **f)** Western blot (Suppl. Fig. 5) shows expression of both TBC1D1 protein isoforms (128 and 138 kDa, respectively) in siLUC-transfected or untreated (ctrl.) C2C12 cells, while only the smaller protein isoform is expressed in TDP-43-silenced cells. The amount of TBC1D1 was normalized against GAPDH or tubulin.

Supplementary Table 1. Splicing-sensitive PCR primers, mouse

Gene	Exon	Transcript	Forward primer 5'-3'	Reverse primer 5'-3'
Poldip3	3	ENSMUST00000058793.13	CATTGGGACTGTAACCCCAG	TGCAAACTTCATCTGCTTGG
Sort1	18 (17b)	ENSMUST00000135636.5	CAGGAGACAAATGCCAAGGT	TGGCCAGGATAATAGGGACA
Rgp1	3	ENSMUST00000030190.8	TGATCGAAGTGGTAGCTGAGC	AGGGTCTAGCCTCAGGTCAC
Pdp1	2	ENSMUST00000108299.1	TGGTGCTGAGTGAGGGAAGGA	TGCTGGCATGGCATCAGAGAAC
Asap2	24	ENSMUST00000064595.14	TGAGACCTATGGAGCCAT	AGTTCCTGATGTCTTAGCCACA
Tmem2	2	ENSMUST00000237802.1	ATGGAGAGGAGATCTGCA	CATACATGATACCCTGTC
Fam220a	2	ENSMUST00000196487.1	ATCGTGGCTTCCATGATG	CTTAAGGCCACATGCTAG
Dnajc5	5	ENSMUST00000116365.8	TCACCTGCTGCTACTGCT	TGGCAGATGCTGGCTGTAT
Nfya	3	ENSMUST00000046719.13	AGCAATAGTTCCACAGAGC	GACACAGGTACTTGCATGAT
Traf7	4	ENSMUST00000088464.11	AGCTGATGGGACTGGCACAT	AGCACAGCTGGCAACACA
Ppfibp1	4	ENSMUST00000136837.1	TCGCAAAGCCAGGTCCTCA	TCTGGAGATGGTGGAGACA
Sapcd2	2	ENSMUST00000028329.12	TGAGCTGTTATGTGTCCCGGT	CAGGCTGCAGTCCACACCATT
Tbc1d1	12 + 13	ENSMUST00000043893.12	AACTCATGCGGTACCACTCC	TGGCCACTCGAAGGAATATC
Tnik	14	ENSMUST00000159680.8	CACTACGAAGAACAGATGCGTC	TCTGCAGTCTTTCTGCTTGTC
Cipc	3	ENSMUST00000185434.6	ATGAAGAAGCTAGCGCGA	CGCTCTTCATGACGACCAT

Supplementary Table 2. Splicing-sensitive PCR primers, human

Gene	Exon	Transcript	Forward primer 5'-3'	Reverse primer 5'-3'
POLDIP3	3	ENST00000252115.10	GCTTAATGCCAGACCGGGAGTTGGA	TCATCTTCATCCAGGTCATATAAATT
PPFIBP1	19	ENST00000228425.11	CTCCATGGATGACAACCCCT	TTTTGGCCGAGAAGAAGCAC
ASAP2	23	ENST00000281419.8	AGCATCTTGCAGAATGAGAC	CCTGATCTGTGAGATCCCA
TRAF7	5	ENST00000564067.5	TACAAGCAGCACTGCAGGA	AGCTGACAGCACAGCTTCA
NFYA	3	ENST00000341376.10	AGTTCGACAGAGCAGATTG	GCATGATGGTTTGACCTTGT
TNIK	14	ENST00000284483.12	CAAAGGCGAGAGAAGGAGCTG	CTGATGCTGAAGGGAAACTAAG
TBC1D1	12 + 13	ENST00000508802.5	AGGTATCACTCAGTGAGC	CTTCACAGGAGTCCCACC

Supplementary Table 3. qPCR primers, mouse

Gene name	Forward primer 5'-3'	Reverse primer 5'-3'
Myog	CAGCCCAGCGAGGGAATTTA	AGAAGCTCCTGAGTTTGCCC
Igf2	CGCTTCAGTTTGTCTGTTCG	AAGCAGCACTCTTCCACGAT
Rfc2	CTGCCGTGGGTTGAAAAATACA	CAGAGGATGCTGGTTGTCTTG
Slmo1	CCACCAATATCACGCTCACGA	CCCCAAGCTAATTCCCTTCACA
Arhgef18	TCAGACAGAAGTGTGGTCCG	GGAGACTGCGAGAGCGAC
Dusp2	ATGGTGGAGATAAGTGCCTGG	GGCTCTGAATCAGGTATGCCA
Gapdh	AGGTCGGTGTGAACGGATTTG	TGTAGACCATGTAGTTGAGGTCA

Supplementary Table 4. Demographic features of muscle donors

Sample no.	Diagnosis	Sex	Age of biopsy	Muscle
1	control	F	52	vastus lateralis
2	control	F	54	vastus lateralis
3	control	M	48	vastus lateralis
4	control	F	22	biceps
5	IBM	M	65	vastus lateralis
6	IBM	M	n.a.	vastus lateralis
7	IBM	F	68	vastus lateralis
8	IBM	M	77	biceps
