# Supplementary material for: Cell environment shapes TDP-43 function with implications in neuronal and muscle disease
Source: Commun Biol. 2022 Apr 5;5:314. doi: 10.1038/s42003-022-03253-8 (PMC8983780; doi:10.1038/s42003-022-03253-8)
Supplement: Supplementary file 3 — Description of Additional Supplementary Files [file 42003_2022_3253_MOESM3_ESM.pdf]

Description of Additional Supplementary Files

File name: Supplementary Data 1
Description: Expression levels of genes assessed by RNA-seq in C2C12 and NSC34 cell lines. Expression level of each gene (reported as its FPKM value), whose expression was assessed by the RNA-seq, is given by experimental group (mean of 3 biological replicates). Groups are labelled as follows: C_si_T43 – C2C12 siTDP, C_Contr – C2C12 siLUC, N_si_T43 – NSC34 siTDP, N_Contr – NSC34 siLUC. The data was used to generate Figs. 1b, 1e, 2b, 5a, 5b and 7b.

File name: Supplementary Data 2
Description: Differentially expressed genes detected by RNA-seq in C2C12 and NSC34 cell lines. Differentially expressed genes identified in C2C12 and NSC34 cell line are reported in two separate excel sheets. For each DEG, its Ensemble gene identifier is followed by adjusted read count in siTDP samples (the mean of 3 biological replicates) and control siLUC samples (the mean of 3 biological replicates), log2-transformed fold change values, the pvalue value, the adjusted p-value that was generated using Benjamini and Hochberg's correction and the official gene name. This data was used to generate Figs. 2a-d and 5c.

File name: Supplementary Data 3
Description: Alternatively spliced genes detected by rMATS in C2C12 and NSC34 cells. Alternatively spliced sequences detected by rMATS following TDP-43 depletion in C2C12 and NSC34 cells. Splicing events detected in each cell line (C2C12 or NSC34, respectively) are reported in separate sheets, classified by event categories (SE – skipped exons, MXE – mutually exclusive exons, A5SS and A3SS – alternative 5' and 3' splice sites, RI – intron retention). Excel tables are self-explanatory. For each splicing event, an Ensamble gene identifier of the host gene is followed by exonic coordinates of the alternative sequence and its flanking exons, inclusion level difference, a p-value and the FDR (false discovery rate). This data was used to generate Figs. 3a and b, 4a and 7c-e.

File name: Supplementary Data 4
Description: Alternatively spliced genes detected by Majiq in C2C12 and NSC34 cells. Alternatively spliced sequences detected by Majiq following TDP-43 depletion in C2C12 and NSC34 cells. Splicing events (i.e., alternatively used junctions) detected in each cell line (C2C12 or NSC34, respectively) are reported in separate sheets. Excel tables are self-explanatory. For each splicing event, an Ensamble gene identifier of the host gene is followed by the official gene name, genomic coordinates of a given alternatively used junction inclusion level difference (ΔPSI) and the FDR (false discovery rate). This data was used to generate Fig. 3c.

File name: Supplementary Data 5
Description: GO terms (category: biological process) enriched by TDP-43- regulated genes in C2C12 and NSC34. GO terms enriched by alternatively spliced or differentially expressed genes identified either in C2C12 or NSC34 cell line are reported in separate excel sheets. For each biological process, its GO identifier is given along with the pathway description, gene ratio and the BG ratio, p-value, the adjusted p-value (q-value), and the list of all genes enriched in a given pathway. This data was used to generate Figs. 2e and f and Fig. 4.

File name: Supplementary Data 6
Description: Inclusion levels of six TDP-43-regulated exons. Inclusion levels of six alternatively spliced exons (as PSI) are given. The table is self-explicatory. This data was used to generate Fig. 9a-c.
