# Supplementary material for: Cell environment shapes TDP-43 function with implications in neuronal and muscle disease
Source: Commun Biol. 2022 Apr 5;5:314. doi: 10.1038/s42003-022-03253-8 (PMC8983780; doi:10.1038/s42003-022-03253-8)
Supplement: Supplementary file 10 — Reporting Summary [file 42003_2022_3253_MOESM10_ESM.pdf]

nature portfolio

Corresponding author(s): Emanuele Buratti

Last updated by author(s): Feb 24, 2022

Reporting Summary

Nature Portfolio wishes to improve the reproducibility of the work that we publish. This form provides structure for consistency and transparency in reporting. For further information on Nature Portfolio policies, see our Editorial Policies and the Editorial Policy Checklist.

Statistics

For all statistical analyses, confirm that the following items are present in the figure legend, table legend, main text, or Methods section.

n/a	Confirmed	
☐	☒	The exact sample size (*n*) for each experimental group/condition, given as a discrete number and unit of measurement
☐	☒	A statement on whether measurements were taken from distinct samples or whether the same sample was measured repeatedly
☐	☒	The statistical test(s) used AND whether they are one- or two-sided<br>*Only common tests should be described solely by name; describe more complex techniques in the Methods section.*
☒	☐	A description of all covariates tested
☐	☒	A description of any assumptions or corrections, such as tests of normality and adjustment for multiple comparisons
☐	☒	A full description of the statistical parameters including central tendency (e.g. means) or other basic estimates (e.g. regression coefficient) AND variation (e.g. standard deviation) or associated estimates of uncertainty (e.g. confidence intervals)
☐	☒	For null hypothesis testing, the test statistic (e.g. *F*, *t*, *r*) with confidence intervals, effect sizes, degrees of freedom and *P* value noted<br>*Give P values as exact values whenever suitable.*
☒	☐	For Bayesian analysis, information on the choice of priors and Markov chain Monte Carlo settings
☒	☐	For hierarchical and complex designs, identification of the appropriate level for tests and full reporting of outcomes
☐	☒	Estimates of effect sizes (e.g. Cohen's *d*, Pearson's *r*), indicating how they were calculated

Our web collection on statistics for biologists contains articles on many of the points above.

Software and code

Policy information about availability of computer code

Data collection	RNA-seq dataset was generated by a commercial provider (Novogene, China).
Data analysis	Spliced Transcripts Alignment to a Reference (STAR) software (v2.5); HTSeq (v0.6.1); R (v3.6.1), DESeq2 R package (v2.1.6.3), replicate multivariate analysis of transcript splicing (rMATS) software (v3.2.1); MAJIQ (v2.1); clusterProfiler package (v3.14.3); Bioconductor mouse annotation package org.Mm.eg.db (v3.10.0).

For manuscripts utilizing custom algorithms or software that are central to the research but not yet described in published literature, software must be made available to editors and reviewers. We strongly encourage code deposition in a community repository (e.g. GitHub). See the Nature Portfolio guidelines for submitting code & software for further information.

Data

Policy information about availability of data

All manuscripts must include a data availability statement. This statement should provide the following information, where applicable:

- Accession codes, unique identifiers, or web links for publicly available datasets
- A description of any restrictions on data availability
- For clinical datasets or third party data, please ensure that the statement adheres to our policy

Datasets generated for this study are deposited in NCBI's Gene Expression Omnibus and are accessible through GEO Series accession number GSE171714

Field-specific reporting

Please select the one below that is the best fit for your research. If you are not sure, read the appropriate sections before making your selection.

☒ Life sciences ☐ Behavioural & social sciences ☐ Ecological, evolutionary & environmental sciences

For a reference copy of the document with all sections, see nature.com/documents/nr-reporting-summary-flat.pdf

Life sciences study design

All studies must disclose on these points even when the disclosure is negative.

Sample size	Each experiment was conduced in 3 independent biological replicates (RNA-seq) following the golden standard of RNA-seq experiments.
Data exclusions	No data was excluded.
Replication	RNA-seq was performed using 3 replicates (see above), while all downstream experiments (RT-PCR, splicing-sensitive PCR, WB) were performed using at least 3 independent biological replicates.
Randomization	The study did not necesitate any randomization procedure.
Blinding	The study did not require any blinding procedure.

Reporting for specific materials, systems and methods

We require information from authors about some types of materials, experimental systems and methods used in many studies. Here, indicate whether each material, system or method listed is relevant to your study. If you are not sure if a list item applies to your research, read the appropriate section before selecting a response.

Materials & experimental systems

n/a	Involved in the study
☐	☒ Antibodies
☐	☒ Eukaryotic cell lines
☒	☐ Palaeontology and archaeology
☒	☐ Animals and other organisms
☒	☐ Human research participants
☐	☒ Clinical data
☒	☐ Dual use research of concern

Methods

n/a	Involved in the study
☒	☐ ChIP-seq
☒	☐ Flow cytometry
☒	☐ MRI-based neuroimaging

Antibodies

Antibodies used	anti-TDP-43 (rabbit, Proteintech, #10782-2-AP); anti-GAPDH (rabbit, Proteintech, #10494-1-AP); anti-HSP70 (rat, EnzoLifeScience, #ADI-SPA-815-J); anti-tubulin (mouse, available inhouse); HRP-conjugated secondary antibodies anti-rabbit (goat, Dako # 0448); anti-mouse (goat, Dako, #P0447); anti-rat (rabbit, Dako, #P0450).
Validation	We used standard commercial antibodies.

Eukaryotic cell lines

Policy information about cell lines

Cell line source(s)	All cell lines were bought from ECACC.
Authentication	We used standard commercial cell lines.
Mycoplasma contamination	Cells used herein were regularly tested for mycoplasma contamination.
Commonly misidentified lines (See ICLAC register)	*Name any commonly misidentified cell lines used in the study and provide a rationale for their use.*

Clinical data

Policy information about clinical studies

All manuscripts should comply with the ICMJE guidelines for publication of clinical research and a completed CONSORT checklist must be included with all submissions.

Clinical trial registration	Clinical data that we investigated herein were generated by the NYGC consortium and are publicly available and the clinical trail was registered by them.
Study protocol	See above.
Data collection	See above
Outcomes	See above.
